# Clinicopathologic characteristics and therapeutic responses of Chinese patients with non-small cell lung cancer who harbor an anaplastic lymphoma kinase rearrangement

**DOI:** 10.1186/s40880-015-0032-8

**Published:** 2015-08-08

**Authors:** Sha Fu, Hai-Yun Wang, Fang Wang, Ma-Yan Huang, Ling Deng, Xiao Zhang, Zu-Lu Ye, Jian-Yong Shao

**Affiliations:** State Key Laboratory of Oncology in South China, Sun Yat-sen University Cancer Center, Collaborative Innovation Center for Cancer Medicine, Guangzhou, Guangdong 510060 P. R. China; Department of Medical Epidemiology and Biostatistics, Karolinska Institutet, 17177 Stockholm, Sweden; Department of Pathology, Sun Yat-sen University Cancer Center, Guangzhou, Guangdong 510060 P. R. China; Department of Molecular Diagnostics, Sun Yat-sen University Cancer Center, 21 Qing Cai Gang Road, Guangzhou, Guangdong 510060 P. R. China

**Keywords:** Anaplastic lymphoma kinase rearrangement, Non-small cell lung cancer, Fluorescence in situ hybridization, Pathology

## Abstract

**Introduction:**

The rearrangement of the anaplastic lymphoma kinase (*ALK*) gene accounts for approximately 1%–6% of lung adenocarcinoma cases and defines a molecular subgroup of tumors characterized by clinical sensitivity to *ALK* inhibitors such as crizotinib. This study aimed to identify the relationship between *ALK* rearrangement and the clinicopathologic characteristics of non-small cell lung cancer (NSCLC) and to analyze the therapeutic responses of crizotinib and conventional chemotherapy to *ALK* rearrangement in NSCLC patients.

**Methods:**

A total of 487 lung cancer patients who underwent testing for *ALK* rearrangement in our department were included in this study. *ALK* rearrangement was examined by using fluorescence in situ hybridization (FISH) assay.

**Results:**

Among the 487 patients, 44 (9.0%) were diagnosed with *ALK* rearrangement by using FISH assay. In 123 patients with adenocarcinoma who were non-smokers and of a young age (≤58 years old), the frequency of *ALK* rearrangement was 20.3% (25/123). Short overall survival (OS) was associated with non-adenocarcinoma tumor type (*P* = 0.006), poorly differentiated tumors (*P* = 0.001), advanced-stage tumors (*P* < 0.001), smoking history (*P* = 0.008), and wild-type epidermal growth factor receptor (*EGFR*) (*P* = 0.008). Moreover, patients with poorly differentiated and advanced-stage tumors had a shorter time to cancer progression compared with those with well differentiated (*P* = 0.023) and early-stage tumors (*P* = 0.001), respectively.

**Conclusions:**

*ALK*-rearranged NSCLC tends to occur in younger individuals who are either non-smokers or light smokers with adenocarcinoma. Patients with *ALK* rearrangement might benefit from *ALK* inhibitor therapy.

## Background

Lung cancer is a lethal but common disease with a 5-year survival rate of approximately 5%–15% [1]. Molecular targeted therapy is emerging as an effective therapeutic strategy for many cancers [2–4]. The following molecularly targeted genes are potentially relevant to oncogenic events and have demonstrated remarkable significance: the v-raf murine sarcoma viral oncogene homolog B1 (*BRAF*), human epidermal growth factor receptor-2 (*HER2*), phosphoinositide 3′-kinase (*PI3K*), v-Ki-ras 2 Kirsten rat sarcoma viral oncogene homolog (*KRAS*), discoidin domain receptor 2 (*DDR2*), epidermal growth factor receptor (*EGFR*), anaplastic lymphoma kinase (*ALK*), c-ros oncogene 1 (*ROS1*), and ret proto-oncogene (*RET*). *ALK* rearrangement represents a new oncogene driver [5, 6].

The echinoderm microtubule-associated protein like 4 (*EML4*)-*ALK* in non-small cell lung cancer (NSCLC) was first discovered as an oncogenic driver gene in 2007; the *EML4*-*ALK* fusion gene was generated by an inversion in the short arm of chromosome 2 [6]. According to the breakpoint on *EML4* (from exon 2 to exon 20), 13 variants of the *EML4*-*ALK* fusion gene have been found [7]. Recently, the TRK-fused gene (*TFG*), the kinesin family member 5B (*KIF5B*), and the kinesin light chain 1 (*KLC1*) were reported to invert and fuse to *ALK* in NSCLC [5, 8, 9].

Compared with other genetic abnormalities in NSCLC, the frequency of the *ALK* rearrangement is approximately 1%–6% in unselected NSCLC [10–12]. Patients with *ALK* rearrangement are highly sensitive to crizotinib, an oral tyrosine kinase inhibitor (TKI) of the c-Met proto-oncogene (*c*-*Met*), *ALK*, and *ROS1* genes. The powerful and specific therapeutic efficacy of this drug on *ALK*-rearranged NSCLC led to the approval by the Food and Drug Administration (FDA) in the United States [13]. Several studies have shown that most of the patients who harbor this chromosomal abnormality have benefited from targeted therapy. A randomized phase III study reported that, in the subgroup of *ALK*-rearranged NSCLC, patients treated with crizotinib showed higher response rate and longer progression-free survival (PFS) compared with those treated with the standard second-line chemotherapies (docetaxel or pemetrexed) [14]. Other studies showed dramatic clinical benefits associated with crizotinib in patients with *ALK*-rearranged NSCLC [15, 16].

The tumors from patients with *ALK* rearrangements are characterized by distinct histologic features including a solid or acinar growth pattern, a cribriform structure, the presence of mucous cells and abundant extracellular mucus, a lack of lepidic growth, and nuclear pleomorphism [17]. Nevertheless, these histologic parameters are of insufficient sensitivity and specificity to detect *ALK* rearrangements, and therefore, histomorphology should not replace confirmatory molecular or immunohistochemical studies [17]. The histomorphology of *ALK*-rearranged lung cancer cannot be used as a screening method. Several methods including reverse transcription-polymerase chain reaction (RT-PCR), fluorescence in situ hybridization (FISH), and immunohistochemistry (IHC) are currently used to identify *ALK* translocations in patients with NSCLC. In the present study, we performed *ALK* FISH in NSCLC cases at the Sun Yat-sen University Cancer Center (SYSUCC) using the gold standard method, Abbott ALK break-apart probe. We analyzed the clinicopathologic features of the patients, their survival status, and the relationship between the clinicopathologic features and *ALK* rearrangement. In previous studies, *ALK* rearrangement represented a unique molecular subset of NSCLC with no overlap with cancers that featured alterations in the *EGFR* or *KRAS* genes [18, 19]. According to *EGFR* and *ALK* status, we retrospectively studied the responses of patients to traditional therapies compared with targeted therapies.

## Patients and methods

### Patient selection

We reviewed 1,000 patients with NSCLC who were observed and tested for *ALK* rearrangement at the Department of Molecular Diagnostics of SYSUCC between February 2012 and November 2013. Patients were involved into this study based on the following criteria: complete clinical data, complete follow-up information, and sufficient paraffin tissue from primary tumors at the time of the initial genetic diagnosis. The patients were excluded if they received any treatment outside of SYSUCC or had a previous history of other cancers that were identified either before or after the NSCLC. Finally, a total of 487 patients were enrolled in this study. All cases were confirmed independently by two experienced pathologists. Pathologic staging was defined according to the International Association for the Study of Lung Cancer (IASLC) TNM staging classification of NSCLC [20]. Histopathologic classification of the cancers was determined according to the 2004 World Health Organization (WHO) histological classification of lung cancer [21]. Patients were classified as non-smokers if they smoked for less than 10 pack-years or smokers if they smoked for 10 pack-years or more in their lifetime. This study was approved by the Institutional Research Medical Ethics Committee of SYSUCC.

### FISH assay

Formalin-fixed, paraffin-embedded, 4-μm sections were used for FISH detection. According to the hematoxylin and eosin stain of the same tissue block, the tumor portion on each slide was selected and demarcated by a single pathologist. The FISH assay included the use of the Vysis LSI ALK Dual Color, Break Apart Probe (Abbott Molecular Inc. Des Plaines, IL, USA), which hybridizes to the 2p23 band with 3′-ALK spectrum orange and 5′-ALK spectrum green. The slides were deparaffinized prior to probe application. Detailed FISH staining procedures have been previously described [22].

FISH signals for each locus-specific FISH probe were assessed under an Olympus BX51 TRF microscope (Olympus, Tokyo, Japan) equipped with a triple-pass filter (DAPI/Green/Orange; Abbott Molecular Inc. Des Plaines, IL, USA). Any tissues with questionable tumor areas were reviewed and noted by a pathologist; the FISH results were evaluated by two independent and experienced pathologists. Cases with *ALK* rearrangements were determined to exhibit one of two patterns: the first type was a classic pattern with one fusion signal (native *ALK*) and two separated orange and green signals; the other was an atypical pattern with one fusion signal (native *ALK*) and an isolated orange signal. By using the signal size as a reference, the tumor cells were considered positive when the probe separation distance in *ALK*-rearranged tumors was larger than the two signal diameters in normal tissue [23]. When *ALK* break-apart signals were found in more than 15% of no less than 50 counted tumor cells, the tumor samples were considered *ALK* rearrangement-positive [23–25]. The tumor samples with a single green signal or an increased copy number of non-rearranged *ALK* genes with fused signals that corresponded to polysomy of chromosome 2 or *ALK* amplification were considered *ALK* rearrangement-negative [23]. For each case, the entire slide was reviewed for possible areas where rearrangements might have been missed.

### *EGFR* mutation

DNA was extracted by a DNA FFPE tissue kit (Qiagen, Valencia, CA, USA) according to the manufacturer’s instructions. DNA was quantified by a NanoDrop 2000 Spectrophotometer (Thermo Fisher Scientific, Waltham, MA, USA), and a total of 200 ng DNA was used for PCR. Nucleotide sequencing of the kinase domain of *EGFR* (exons 18, 19, 20, and 21) was performed with PCR amplification and Sanger sequencing. PCR-amplified products were purified with a PCR purification kit (Qiagen, Valencia, CA, USA) and were sequenced with a BigDye Terminator Cycle Sequencing Reaction Kit and an ABI 3500 XL Genetic Analyzer (Hitachi, Tokyo, Japan).

### Follow-up and classification of cause of death

The clinical data including follow-up information were obtained from the medical record system as well as by telephone interview. The collection of follow-up data for each case was executed based on a semi-annual schedule. For deceased patients, we classified the underlying cause of death as stated on the death certificate. Local relapse was defined as recurrence of lung cancer as determined by biopsy, whereas distant metastasis was defined according to evidence shown on a chest computed tomography (CT) scan, abdominal ultrasound, or bone scan. The overall survival (OS) was defined as the period from the date of diagnosis to death from any cause or the date of last contact; the PFS was defined as the period from the date of diagnosis to the first regional or distant metastasis. The last follow-up was performed in April 2014, and the median follow-up was 18.8 months (range 0.3–172.2 months). In total, 86 (17.7%) cases died during follow-up.

### Statistical analysis

The relationship between *ALK* status and the clinicopathologic features was analyzed by using Chi square test and Fisher’s exact test. Kaplan–Meier curves with the log-rank test were applied for OS and PFS analysis. Cox proportional hazards models were used to compare independent predictive factors of each biological and clinicopathologic feature. A two-sided statistical significance was defined as *P* < 0.05. All analyses were performed with SPSS 16.0 Statistics software (SPSS Inc., Chicago, IL, USA).

## Results

### Clinicopathologic characteristics

Of the 487 patients, 133 (27.3%) underwent biopsies, and 354 (72.7%) underwent surgical resections. Briefly, 303 (62.2%) were males, and 184 (37.8%) were females, with a median age of 58 years (range 25–86 years). The histological types included adenocarcinomas (78.4%, 382/487) and non-adenocarcinomas (21.6%, 105/487); the 105 non-adenocarcinomas comprised 73 squamous carcinomas, 25 large cell carcinomas, and 7 sarcomatoid carcinomas. The clinicopathologic characteristics of the 487 patients with lung cancer are listed in Table 1.

**Table 1:** Associations between *ALK* rearrangement and the clinicopathologic characteristics of 487 patients with non-small cell lung cancer (NSCLC)

Characteristic	Number of patients (%)	*ALK* rearrangement [cases (%)]		*P*
		Negative	Positive	
Total	487	443 (91.0)	44 (9.0)	
Age				
≤58 years	261 (53.6)	229 (51.7)	32 (72.7)	0.008
>58 years	226 (46.4)	214 (48.3)	12 (27.3)	
Sex				
Male	303 (62.2)	279 (63.0)	24 (54.5)	0.271
Female	184 (37.8)	164 (37.0)	20 (45.5)	
Histological type				
Adenocarcinoma	382 (78.4)	339 (76.5)	43 (97.7)	0.001
Others	105 (21.6)	104 (23.5)	1 (2.3)	
Differentiation				
Well	206 (42.3)	187 (42.2)	19 (43.2)	0.901
Poor	281 (57.7)	256 (57.8)	25 (56.8)	
Clinical stage				
I + II	226 (46.4)	213 (48.1)	13 (29.5)	0.019
III + IV	261 (53.6)	230 (51.9)	31 (70.5)	
Smoking history				
Non-smoker	239 (49.1)	207 (46.7)	32 (72.7)	0.001
Smoker	248 (50.9)	236 (53.3)	12 (27.3)	
*EGFR* mutation status				
Negative	328 (67.4)	285 (64.3)	43 (97.7)	<0.001
Positive	159 (32.6)	158 (35.7)	1 (2.3)	

*ALK* anaplastic lymphoma kinase, *EGFR* epidermal growth factor receptor.

All values are presented as number of patients followed by percentages in the parentheses.

### Detection of *ALK* rearrangement by FISH

Of the 487 NSCLC cases, 44 (9.0%) were identified with *ALK* rearrangements. The percentage of *ALK* break-apart signals ranged from 16% to 90%. Of the 44 *ALK*-rearranged cases, 15 (34.1%) showed more than 50% split fluorescence signals, and 29 (65.9%) demonstrated less than 50% split signals. The mean percentage of positive nuclei was 51.7% in the cases with *ALK* rearrangement. Among the 44 patients with *ALK* rearrangement, the median age was 49 years (range 25–80 years). We compared the clinicopathologic characteristics between the *ALK*-fusion-positive group and the *ALK*-fusion-negative group and found significant differences in age (*P* = 0.008), histological type (*P* = 0.001), clinical stage (*P* = 0.019), smoking history (*P* = 0.001), and *EGFR* mutation status (*P* < 0.001); no significant difference was found in other clinicopathologic parameters between the two groups (Table 1).

**Fig. 1:**
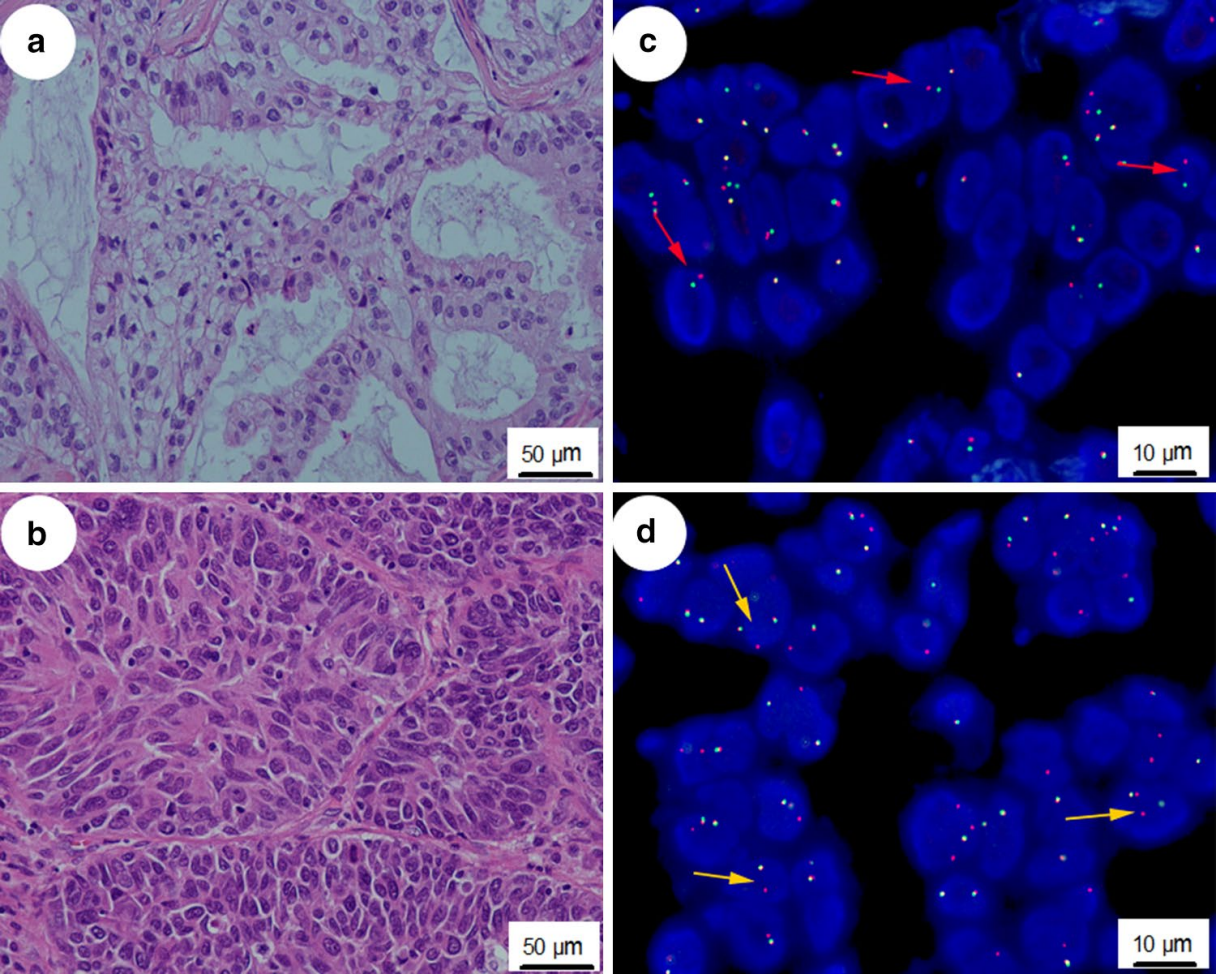
Representatives of hematoxylin–eosin (H&E) staining and fluorescence in situ hybridization (FISH) in non–small cell lung cancer (NSCLC) lesions with anaplastic lymphoma kinase (*ALK*) rearrangement. **a** An *ALK*-rearranged adenocarcinoma with a mucinous pattern by H&E staining (×200). **b** An *ALK*-rearranged squamous carcinoma with a solid growth pattern by H&E staining (×200). **c** *ALK* break-apart signals by FISH with a split orange (staining 3′-2 chromosomal/DNA region) and green (staining 5′-2 chromosomal/DNA region) signal pattern (*red arrows*), indicating an *ALK* rearrangement-positive status. **d** *ALK* break-apart signals by FISH with an isolated orange signal pattern (*yellow arrows*), indicating an *ALK* rearrangement-negative status.

Of the 44 patients with *ALK* rearrangement, 43 (97.7%) had adenocarcinomas, and 1 (2.3%) had squamous carcinoma (Fig. 1). Of the 43 cases of adenocarcinomas, 30 (69.8%) were typical adenocarcinomas, 6 (14.0%) were mucinous adenocarcinomas, and 3 (7.0%) were papillary adenocarcinomas. Intriguingly, we found 4 *ALK*-rearranged adenocarcinomas with morphologic features of focal squamous differentiation. The predominant components of typical adenocarcinomas were as follows: acinar growth pattern in 25 cases, solid growth pattern in two cases, and mixed growth pattern in three cases.

### Analysis of *EGFR* mutation status and clinical characteristics in patients with NSCLC

Of the 487 patients, we identified 159 (32.6%) with *EGFR* mutations in exons 18, 19, 20, and 21. Of the 159 cases, 68 (42.8%) were determined to have an exon 19 deletion, 3 (1.9%) had an exon 20 mutation, and 87 (54.7%) had an exon 21 mutation. We found that 1 (0.6%) case exhibited double mutations (15 bp deletion and L858R). However, no mutations were detected in exon 18. The predominant types of mutations were found in exons 19 and 21, which accounted for 97.5% of the mutations. Significant differences were shown in age (*P* = 0.030), sex (*P* < 0.001), histological type (*P* < 0.001), differentiation (*P* < 0.001), and smoking history (*P* < 0.001) between the *EGFR* mutation-negative group and the *EGFR* mutation-positive group. However, no significant difference was observed in other clinical characteristics (Table 2).

**Table 2:** Associations between *EGFR* mutation status and the clinicopathologic features of patients with NSCLC

Characteristic	*EGFR* mutation [cases (%)]		*P*
	Negative	Positive	
Total	328 (67.4)	159 (32.6)	
Age			
≤58 years	187 (57.0)	74 (46.5)	0.030
>58 years	141 (43.0)	85 (53.5)	
Sex			
Male	231 (70.4)	72 (45.3)	<0.001
Female	97 (29.6)	87 (54.7)	
Histological type			
Adenocarcinoma	226 (68.9)	156 (98.1)	<0.001
Others	102 (31.1)	3 (1.9)	
Differentiation			
Well	118 (36.0)	88 (55.3)	<0.001
Poor	210 (64.0)	71 (44.7)	
Clinical stage			
I + II	153 (46.6)	73 (45.9)	0.879
III + IV	175 (53.4)	86 (54.1)	
Smoking history			
Non-smoker	130 (39.6)	109 (68.6)	<0.001
Smoker	198 (60.4)	50 (31.4)	

All values are presented as number of patients followed by percentages in the parentheses. Other footnotes as in Table 1.

### Survival analysis of NSCLC patients

**Fig. 2:**
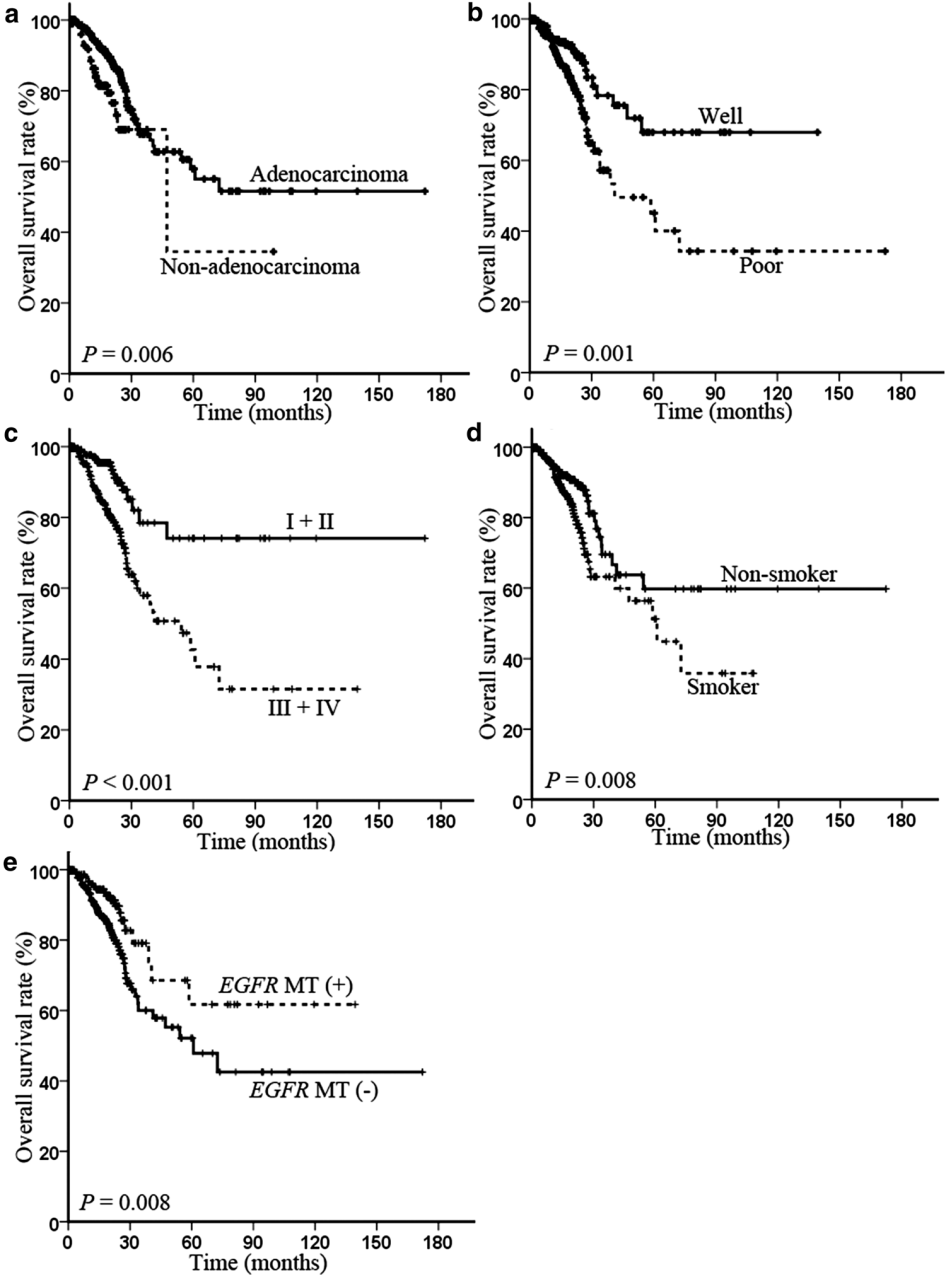
Kaplan–Meier overall survival curves stratified by different clinicopathologic parameters in NSCLC patients. **a** Histological type; **b** differentiation; **c** clinical stage; **d** smoking history; **e** epidermal growth factor receptor (*EGFR*) mutation (MT) status.

**Fig. 3:**
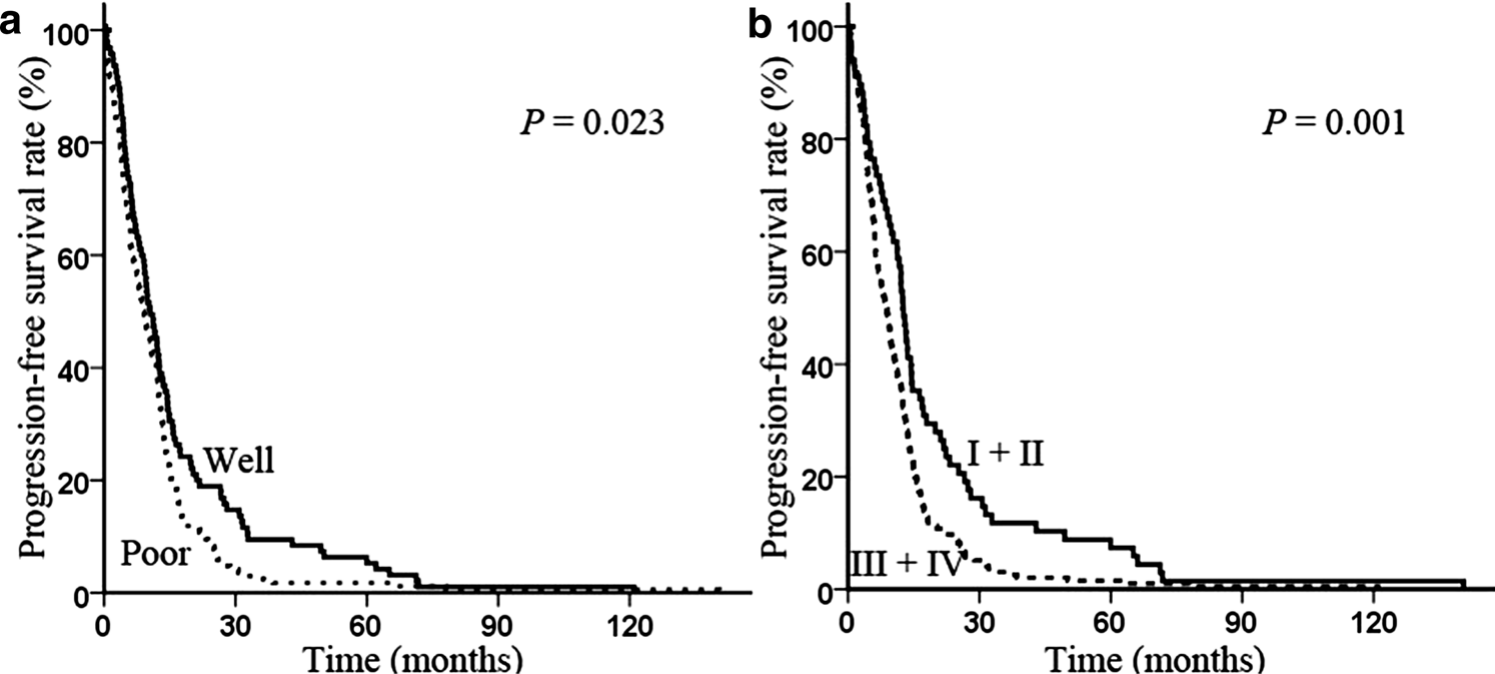
Kaplan–Meier progression-free survival curves stratified by different clinicopathologic parameters in NSCLC patients. **a** Differentiation; **b** clinical stage.

Using the Kaplan–Meier estimate method, we found that the OS was significantly associated with histological type (*P* = 0.006), differentiation (*P* = 0.001), clinical stage (*P* < 0.001), smoking history (*P* = 0.008), and *EGFR* mutation status (*P* = 0.008) (Fig. 2). However, the OS showed no significant association with age (*P* = 0.794), sex (*P* = 0.144), or *ALK* rearrangement status (*P* = 0.300) (data not shown). On the contrary, patients with poorly differentiated tumors and those with advanced-stage tumors had a shorter time to cancer progression compared with those with well differentiated tumors (*P* = 0.023) and those with early-stage tumors (*P* = 0.001) (Fig. 3). The PFS showed no significant association with age (*P* = 0.293), sex (*P* = 0.958), histological type (*P* = 0.099), smoking history (*P* = 0.442), *ALK* rearrangement status (*P* = 0.212), or *EGFR* mutation status (*P* = 0.464) (data not shown).

Several variables including age, sex, histological type, differentiation, clinical stage, smoking history, tumor status, lymph node status, metastasis status, *EGFR* mutation status, and *ALK* rearrangement status were analyzed by using univariate and multivariate Cox proportional hazard regression analyses. The univariate analysis results showed that the OS significantly associated with histological type [hazard ratio (HR) 1.971, 95% confidence interval (CI) 1.200–3.235, *P* = 0.007], differentiation (HR 0.449, 95% CI 0.279–0.721, *P* = 0.001), clinical stage (HR 3.091, 95% CI 1.855–5.149, *P* < 0.001), smoking history (HR 1.779, 95% CI 1.153–2.747, *P* = 0.009), T category (HR 2.475, 95% CI 1.605–3.816, *P* < 0.001), N category (HR 3.155, 95% CI 1.855–5.366, *P* < 0.001), M category (HR 2.275, 95% CI 1.477–3.503, *P* < 0.001), and *EGFR* mutation (HR 0.509, 95% CI 0.306–0.848, *P* = 0.009). The multivariate analysis results showed that histological type (HR 1.926, 95% CI 1.102–3.364, *P* = 0.021), N category (HR 2.403, 95% CI 1.166–4.956, *P* = 0.018), and M category (HR 2.164, 95% CI 1.292–3.624, *P* = 0.003) were independent prognostic factors for OS in NSCLC (Table 3). According to univariate analysis results, PFS were significantly associated with differentiation (HR 0.744, 95% CI 0.576–0.961, *P* = 0.023), clinical stage (HR 1.595, 95% CI 1.202–2.116, *P* = 0.001), T category (HR 1.582, 95% CI 1.218–2.055, *P* = 0.001), N category (HR 1.438, 95% CI 1.071–1.931, *P* = 0.016), and M category (HR 1.576, 95% CI 1.214–2.046, *P* = 0.001). Further multivariate analysis indicated that T category (HR 1.499, 95% CI 1.144–1.964, *P* = 0.003) and M category (HR 1.477, 95% CI 1.105–1.974, *P* = 0.008) were independent predictors of PFS (Table 3).

**Table 3:** Cox regression analysis of OS and PFS in 487 patients with NSCLC

Variable	Subset	OS		PFS	
		HR (95% CI)	*P*	HR (95% CI)	*P*
Univariate analysis					
Age	≤58 vs. >58	1.058 (0.692–1.618)	0.794	0.876 (0.685–1.121)	0.294
Sex	Male vs. female	0.710 (0.448–1.126)	0.146	1.007 (0.781–1.299)	0.958
Histological type	Adenocarcinoma vs. others	1.971 (1.200–3.235)	0.007	1.294 (0.951–1.761)	0.101
Differentiation	Well vs. poor	0.449 (0.279–0.721)	0.001	0.744 (0.576–0.961)	0.023
Clinical stage	I + II vs. III + IV	3.091 (1.855–5.149)	<0.001	1.595 (1.202–2.116)	0.001
Smoking history	Non-smoker vs. smoker	1.779 (1.153–2.747)	0.009	1.100 (0.862–1.403)	0.443
T category	T1–2 vs. T3–4	2.475 (1.605–3.816)	<0.001	1.582 (1.218–2.055)	0.001
N category	N0 vs. N1–3	3.155 (1.855–5.366)	<0.001	1.438 (1.071–1.931)	0.016
M category	M0 vs. M1	2.275 (1.477–3.503)	<0.001	1.576 (1.214–2.046)	0.001
*EGFR* mutation	Negative vs. positive	0.509 (0.306–0.848)	0.009	0.845 (0.647–1.102)	0.213
*ALK* rearrangement	Negative vs. positive	1.397 (0.740–2.635)	0.302	1.155 (0.785–1.701)	0.465
Multivariate analysis					
Histological type	Adenocarcinoma vs. others	1.926 (1.102–3.364)	0.021	–	–
T category	T1–2 vs. T3–4	–	–	1.499 (1.144–1.964)	0.003
N category	N0 vs. N1–3	2.403 (1.166–4.956)	0.018	–	–
M category	M0 vs. M1	2.164 (1.292–3.624)	0.003	1.477 (1.105–1.974)	0.008

*OS* overall survival, *PFS* progression-free survival, *HR* hazard ratio, *CI* confidence interval. Other footnotes as in Table 1.

### Therapeutic responses and clinical outcome of patients with *ALK* rearrangement

Of the 44 patients with *ALK* rearrangement, 18 (40.9%) were treated with crizotinib, 4 (9.1%) were treated with an EGFR-TKI, and 22 (50.0%) were treated with conventional chemotherapy. After treated with crizotinib, the tumors in the 18 patients shrank, and both the symptoms and the quality of life were substantially improved. By the last follow-up date, the median OS in the 18 patients with *ALK* rearrangements who were treated with an ALK-TKI was higher than that in the 22 patients with *ALK* rearrangements who were treated with conventional chemotherapy, but there was no statistical difference (21.2 vs. 19.1 months, *P* = 0.587). Because of the short follow-up time, no significant difference was found between the two groups in terms of OS rate (*P* = 0.773) or PFS rate (*P* = 0.608).

## Discussion

The identification of *ALK* rearrangements in NSCLC is pivotal to guide the appropriate treatment with ALK-TKI. In this study, we used an ALK break-apart probe assay to test 487 NSCLC cases and identified 44 (9.0%) with *ALK* rearrangements, which was in accordance with the results of previous investigations [25–28]. The frequency of *ALK* rearrangements was 20.3% (25/123) in young patients with the adenocarcinoma subtype who were non-smokers, which was also consistent with the previously reported results [24, 27, 29, 30]. Our study also revealed other characteristics of NSCLC with *ALK* rearrangements, such as advanced stage (70.5%, 31/44) and wild-type *EGFR* (97.7%, 43/44). Additionally, patients with advanced-stage tumors and poorly differentiated tumors had a shorter time to cancer progression in comparison with those with early-stage tumors and well differentiated tumors.

Tumors with *ALK* rearrangement were found to exhibit papillary and acinar growth patterns [18], mucinous cribriform patterns [17], and a solid signet-ring cell pattern [24]. In addition, the coexistence of glandular and squamous morphologies was also reported in previous studies [18, 24]. In our study, acinar, papillary, and mucinous adenocarcinomas were found in *ALK*-rearranged cases. Interestingly, in our study, we also detected *ALK* rearrangements in a case of squamous cell carcinoma. Furthermore, 4 *ALK*-rearranged adenocarcinomas were observed in patients with focal squamous differentiation. This suggests that a test for *ALK* rearrangement should be performed in patients with classical morphologic patterns, mixed squamous and adenocarcinoma patterns as well as in patients with squamous carcinoma in case of a wrong therapeutic decision.

*ALK* rearrangement was generally reported to be found in tumors with wild-type *EGFR* and *KRAS* [7, 19, 27, 31, 32]. However, some investigators also observed the coexistence of *ALK* rearrangement and *EGFR* mutations [33–35]. It was reported that patients who harbor a concurrent *EGFR* mutation and an *ALK* rearrangement may partially respond to *EGFR* inhibitors [36–38]. Interestingly, we identified one patient with a concomitant *ALK* rearrangement and *EGFR* mutation who had undergone therapy with erlotinib. Nevertheless, this patient experienced disease progression less than 1 month later as confirmed by CT scan. The mechanism of resistance might be affected by different signal transduction pathways. Sasaki *et al*. [38] reported that in in vitro studies the co-expression of an *EGFR* mutation and the *EML4*-*ALK* fusion gene might lead to resistance to targeted therapies. It is therefore important to determine the optimal combination of a given EGFR-TKI and *ALK* inhibitor or other new therapeutic regimens for patients with a concomitant *EGFR* mutation and an *ALK* rearrangement.

In an analysis of OS and PFS between *ALK* rearrangement-positive and *ALK* rearrangement-negative groups, no statistical significance was observed. The reason might be that the patients with *ALK* rearrangements, in our study, were not treated with targeted therapy or they were treated for only a short time. Li *et al*. [39] reported that *EGFR*/*KRAS* mutation status appeared to be significantly associated with neither PFS nor OS if these patients did not receive targeted therapies. This hypothesis is consistent with the results of our study. All evidence supports the hypothesis that gene subtypes could only predict the therapeutic response but not the survival benefit until patients received a given molecular targeted therapy.

Patients with *EGFR* mutations demonstrated a superior PFS after treatment with molecular targeted therapy compared with those treated with traditional platinum-doublet chemotherapy [40]. However, in our study, disease progression was observed in two patients with *ALK* rearrangement after they were treated with crizotinib, and the two patients were considered resistant to crizotinib. Presently, the mechanism of resistance to *ALK* inhibitors is thought to be a compensatory mechanism or the occurrence of drug-resistant gene mutation, such as a mutation in *ALK*, *EGFR*, *KRAS*, or *BRAF* [41, 42]. Rapid disease progression can deprive the patients of a second chance for survival. Spaans *et al*. [42] suggested that a combination targeted therapy that simultaneously inhibits multiple resistance pathways would elicit a better clinical response. However, the toxicity of combination drugs requires further investigation.

There are some limitations in our study. First, this was a retrospective study and all cases were selected within a single hospital. Second, many patients with *ALK* rearrangement or *EGFR* mutations could not receive targeted therapy or they had a short medication duration due to budget limitations. Therefore, we were not able to exclude geographic and demographic variations that might have influenced the outcome of the study.

In conclusion, the patients who harbor *ALK* rearrangements tend to be relatively young, non-smokers or light smokers with the adenocarcinoma subtype. Prior to treatment, it is necessary to assess *ALK* rearrangement status to determine the appropriate therapeutic regimen. Because the underlying mechanism of the partial response to targeted therapy is unknown, we will continue to investigate this mechanism in patients with NSCLC in the future.
